# 66 The Association of Burn Size and Global Functioning: A Preschool LIBRE1-5 Study

**DOI:** 10.1093/jbcr/irac012.069

**Published:** 2022-03-23

**Authors:** Kate E Surette, Pengsheng Ni, Khushbu F Patel, Silvanys L Rodríguez-Mercedes, Camerin A Rencken, Renata Fabia, Carrie Tully, Tina L Palmieri, Kathleen S Romanowski, Petra Warner, Frederick J J Stoddard, Jeffrey C Schneider, Lewis E Kazis, Colleen M Ryan

**Affiliations:** Shriner's Hospitals for Children - Boston, Boston, Massachusetts; Boston University School of Public Health, Boston, Massachusetts; Shriners Hospitals for Children - Boston/MGH, Boston, Massachusetts; Shriners Hospitals for Children - Boston, Boston, Massachusetts; Shriners Hospitals for Children - Boston, Seattle, Washington; Nationwide Children's Hospital, Columbus, Ohio; Children's National Hospital, Washington, District of Columbia; UC Davis and Shriners Hospitals for Children Northern California, Sacramento, California; University of California, Davis and Shriners Hospitals for California Northern California, Sacramento, California; Shriners Children's Ohio, Dayton, Ohio; Harvard Medical School, Boston, Massachusetts; , Massachusetts; Boston University School of Public Health, Spaulding Rehabilitation Hospital, Harvard Medical School, Boston, Massachusetts; Harvard Medical School, Boston, Massachusetts

## Abstract

**Introduction:**

Between the ages of one and five, children gain increased mobility and begin to explore their surroundings. This makes them a particularly vulnerable age group for burn-related injuries, which can influence a child’s physical and psychosocial development. Previous research in adult burn survivors associated larger burn size with poorer functional outcomes for social activities. Currently, there are limited data on the association in preschool aged survivors between burn size and functioning. The aim of this study is to understand how demographic characteristics, particularly burn size, correlate with the global functioning items using data from the Preschool-LIBRE_1-5_ study.

**Methods:**

The Preschool-LIBRE_1-5_ was field-tested with 426 parents of burn survivors. Eight global items assessed change in functioning in four domains (physical, psychological, communication & language, and social) compared to pre-burn functioning. Demographic variables included gender, race, age at survey completion, total body surface area burned (TBSA), ethnicity, and pain severity. Post-burn abilities were assessed with “Following the burn injury, my child lost abilities he/she had before the burn injury in…”, measured with a yes or no response and compared to other children without burns with, “Compared to other children in the same age, in general, how would you rate your child’s…”, measured with a 5-point Likert scale ranging from much worse to much better. Multivariate logistic regression with multiple imputation for missing values were used to measure the association between demographic characteristics and global items.

**Results:**

The population had a mean age at time of burn injury of 1.9 + 1.1 years and mean TBSA% of 4.2 + 8.0. Of the 426 participants, 305 have a TBSA < 5%, 45 have a TBSA between 5%-15% and 45 have a TBSA >15%. Larger TBSA was associated with lower odds of abilities in functional status for all four global functioning items. Adjusted odd ratios with 95% CI’s included communication and language 0.57(0.35,0.93), physical function 0.55(0.37,0.83), social function 0.33(0.2,0.52), and psychological/behavioral function 0.49(0.31,0.75). There was also a negative correlation of larger TBSA with weaker social abilities of the child compared to other children without burns.

**Conclusions:**

The findings of this study show a negative association between a child’s burn size and parent-reported functioning in the four domains post-burn injury. These findings may help clinicians improve pediatric recovery and rehabilitation.

